# Range of effectiveness of hydraulic diameter model as an analytical solution for rectangular microchannels

**DOI:** 10.1016/j.heliyon.2024.e41498

**Published:** 2024-12-26

**Authors:** Koichiro Kobayashi, Kenji Sakamoto

**Affiliations:** a Shipping Technology Department, National Institute of Technology, Oshima College, Suo-Oshima, 742-2193, Japan; b Department of Intelligent and Control Systems, Kyushu Institute of Technology, Iizuka, Fukuoka, 820-8502, Japan

**Keywords:** Microfluidic channel, Capillary flow, Rectangular channel, Hydraulic diameter

## Abstract

Spontaneous capillary flow in rectangular microfluidic channels is employed in microfluidic devices for various applications. The exact solution for flow in a rectangular cross-sectional channel has a complex point that contains an infinite sum term. The flow depends on the depth-width ratio of the rectangular channel's cross-section, *ε*. In previous studies, several approximations from exact solutions were useful for *ε* values smaller than 1 or 2. In this study, we propose a conversion equation J(ε) that turns the hydraulic-diameter (HD) model into a rectangular-channel (RC) model. Experiments: Rectangular type flow experiments with ethanol solution were conducted to confirm the difference between the RC and HD models when the analysis was performed based on each model. Findings: J(ε) depends on ε. At ε=0.441,2.27, the two models coincide. For 0.247<ε<4.04, J(ε) deviates by ±12.5%.

## Graphical abstract

**Figure:**
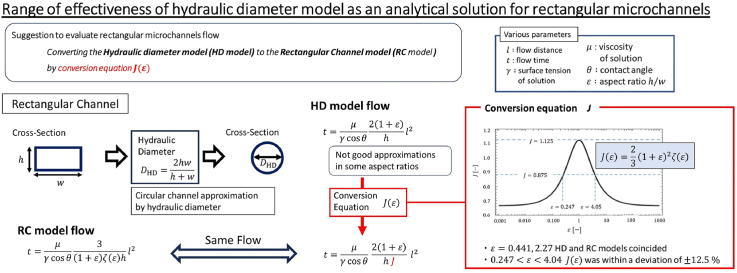

## 1 Introduction

Spontaneous capillary-driven flow in microfluidic channels enables low-cost sample flow without the need for pumps or syringes. Scholars have employed this simple fluid delivery system for microfluidic devices in biotechnology and medical applications, including medical diagnostic microfluidic devices [[1], [2], [3]].

For example, one-step immunoassays were developed on a microfluidic chip with integrated reagents [4], where the capillary force acting on the micropillars powered the immunoassay device [5]. Similarly, an immunoassay for C-reactive protein was developed using capillary flow with an array of microchannels [6]. Microstructures were also used for the patterned delivery of immunoglobulins in microfluidic channels [7]. The capillary drive was further employed in allergy testing using a micro-total analysis system [[8], [9], [10]]. Additionally, viscosity measurement chips for body fluid samples were investigated by analyzing the capillary flow [11].

The fluid mechanics of cylindrical microchannels have been extensively analyzed using the Lucas–Washburn–Rideal (LWR) equation [[12], [13], [14]]. The solution is calculated based on atmospheric, hydrostatic, and capillary pressures. Bousanquet [15] further included an inertial term. Similarly, Qu$\overset{´}{e}$r$\overset{´}{e}$ [16] studied capillary rise, including inertial forces, and discovered that oscillations occurred when the fluid viscosity was lower than a certain range. Das et al. [17] studied the capillary flow in the initial stage leading up to the LWR equation. For a flow with gravitational effects, Fries and Dreyer [18,19] proposed an analytical solution for the capillary rise owing to hydrostatic pressure.

Rectangular channels are frequently used in real microfluidic devices owing to their simplicity. Jong et al. [20] considered capillary imbibition in rectangular microchannels. Brody et al. [21] and Ichikawa et al. [22] derived analytical solutions for a flow in rectangular microchannels. In a similar context, Ouali et al. [23] considered the effect of gravity. However, these solutions for rectangular channels have a complex point that contains an infinite sum term. In a rectangular channel, *h* and *w* indicate the depth and width of its cross-section, respectively, and $\varepsilon =\frac{h}{w}$ denotes their aspect ratio. Several previous studies use approximations in an infinite sum term. Bruus [24] and Zhu et al. [25] presented approximations that assume that *ε* is less than 1. Ouali et al. [23] introduced an approximation that is used with *ε* values between 0 and 2. Consequently, the range of adaptation for *ε* is limited in the aforementioned studies. Recently, in research on capillary force flow, Yelkhovsky et al. [26] investigated the two-phase flow of gas and liquid in polygonal channels, Anoop et al. [27] examined the effect of deformation of rectangular channels, Testoni et al. [28] proposed a model for the capillary rise of liquid in flax fiber by applying the LWR equation, and Gurumurthy et al. [29] studied the capillary rise of liquid in open rectangular channels.

Other researchers have simplified pressure-drop calculations in a rectangular channel by simply replacing the pressure drop term. Hydraulic diameters (HDs) that approximate circular tube channels based on the dimensions of the channel cross-section were used in flow calculations. The approach was applied to tubes with various cross-sections (Shah et al. [30], and Ma [31]). Furthermore, Bahrami et al. [32] and Muzychka and Yovanovich [33] proposed an approximation method using the square root of a channel's cross-section area as the characteristic length scale for a rectangular channel. Berthier et al. [34] proposed a model that uses friction length to derive information on the cross-section of a flow path. For a specific *ε*, these approaches were evaluated by comparing the theoretical results with flow experiments in channels with cross-sections, including rectangular types.

As rectangular microchannels are often used for ease of construction, we focused on adapting the HD model to rectangular microchannels. We studied the deviation from exact solutions of the HD model in channel cross-sections with aspect ratios ranging between 0.001 *< ε <* 1000 and discussed the range of aspect ratios in which the HD model can be adapted. We compared the extended solution of rectangular channels using the HD in the LWR solution with the analytical solution of the rectangular channel by Ouali et al. [23]. The theoretical models of liquid penetration in a rectangular channel were reviewed, including the HD model, which is based on the HD, and the exact solution of the rectangular channel (RC) model, which is based on the balance of forces. Converting the HD model to the RC model, we propose a conversion equation that depends on the depth-width ratio of the cross-section of a microchannel. We employ an approximation method to analyze the equation. The dependence on the depth-width ratio in the cross-section of the microchannel is discussed considering the relationship between the solutions of the two models. To verify these dependencies, experiments were conducted using flowing ethanol solutions in rectangular microfluidic channels (values of aspect ratio *ε* = 0.167*,*0.5*,*1.0*,*2.5).

## 2 Theory—Models of liquid penetration in channels

### 2.1 HD and RC models

A rectangular channel was replaced with a cylindrical channel using the concept of HD; radius $r_{\text{HD}}$, which is defined by the cross-sectional area $S_{RC}=hw$ and circumference $L_{\text{RC}\;}=2\left(h+w\right)$ of the rectangular channel as

(1) $$\begin{array}{c} r_{\text{HD}}=\frac{2S_{RC}}{L_{RC}}=\frac{h}{1+\varepsilon } \end{array}$$

was used to rewrite the cylindrical channel equation into the HD model.

To compare the HD and RC models, we introduce a theoretical model of liquid penetration *l* over time *t*. The HD model is based on the analytical solution of flow in a cylindrical channel [[12], [13], [14],^18^,^19^]. The theoretical model of a cylindrical channel of radius *r* with a quasi steady state flow of the liquid meniscus on the horizontal plane was considered to investigate the balance of capillary and viscous forces. The LWR equation is

(2) $$\begin{array}{c} t=\frac{2\mu }{r\gamma \;\cos\;\theta }l^{2}\text{,} \end{array}$$

where γ indicates the surface tension, μ denotes the viscosity of the liquid, and θ denotes the contact angle. Using the radius from the HD, the distance traveled by the liquid meniscus is calculated by replacing $r\to r_{HD}=\frac{h}{1+\varepsilon }$ in Eq. (2) with Eq. (1), as

(3) $$\begin{array}{c} t=\frac{2\mu \left(1+\varepsilon \right)}{h\gamma \;\cos\;\theta }l^{2}\text{.} \end{array}$$

The RC model is the analytical solution based on the balance of forces in a steady flow [[21], [22], [23]].

(4) $$\begin{array}{c} t=\frac{3}{\left(1+\varepsilon \right)\zeta \left(\varepsilon \right)h}\frac{\mu }{\gamma \;\cos\;\theta }l^{2}\text{,} \end{array}$$

where *ζ*(*ε*) is given by

(5) $$\begin{array}{c} \zeta \left(\varepsilon \right)=1-6\varepsilon \lim_{N\to \infty }\sum_{k=0}^{N}{\left\{\frac{2}{\left(2k+1\right)\pi }\right\}}^{5}\;\tanh\left[\frac{\left(2k+1\right)\pi }{2\varepsilon }\right] \end{array}.$$

### 2.2 Comparison of the HD and RC models in a rectangular channel

#### 2.2.1 Comparison equation

The ratio of each force in the HD model is compared with that in the RC model. The capillary force is the force that promotes liquid penetration and is dependent on the circumference $L_{c}$ of the channel cross-section, as represented by $f_{c\text{ap}}=L_{c}\gamma \;\cos\;\theta$. The ratio related to the capillary force of the HD model $f_{\text{cap},\text{HD}}=2\pi \frac{1}{1+\varepsilon }h\gamma \;\cos\;\theta$ and the RC model $f_{\text{cap},\text{RC}}=2\pi \frac{1+\varepsilon }{\varepsilon }h\gamma \;\cos\;\theta$ is represented by $K_{1}$

(6) $$\begin{array}{c} K_{1}=\frac{f_{\text{cap},\text{HD}}}{f_{\text{cap},\text{RC}}}=\frac{\pi \varepsilon }{{\left(1+\varepsilon \right)}^{2}}\text{.} \end{array}$$

The viscous force is the force that prevents liquid penetration. It is the sum of the shear stress acting on the inner surface of the channel and is proportional to the liquid tip travel distance l and velocity $\frac{dl}{dt}$, as represented by $f_{\mathrm{visc}}=-al\frac{dl}{dt}$. Here, a is a proportionality constant. The ratio related to the viscous force of the HD model $f_{\text{visc},\text{HD}}=-8\pi \mu l\frac{dl}{dt}$ and the RC model $f_{\text{visc},\text{RC}}=-\frac{12\mu }{\varepsilon \zeta \left(\varepsilon \right)}l\frac{dl}{dt}$ is represented by *K*_2_

(7) $$\begin{array}{c} K_{2}=\frac{f_{\text{visc},\text{HD}}}{f_{\text{visc},\text{RC}}}=\frac{2}{3}\pi \varepsilon \zeta \left(\varepsilon \right)\text{.} \end{array}$$

Assuming quasi steady state flow in a rectangular channel, the equation of force balance based on the RC model is given as

(8) $$\begin{array}{c} f_{c\text{ap},\text{RC}}+f_{v\text{isc},\text{RC}}=0\text{.} \end{array}$$

Substituting Eqs. (6), (7) into Eq. (8) while using the forces in the HD model yields

(9) $$\begin{array}{c} Jf_{\text{cap},\text{HD}}+f_{\text{visc},\text{HD}}=0\text{,} \end{array}$$

where *J* is the conversion equation between the HD model and the RC model, which is written as

(10) $$\begin{array}{c} J\left(\varepsilon \right)=\frac{K_{2}}{K_{1}}=\frac{2{\left(1+\varepsilon \right)}^{2}\zeta \left(\varepsilon \right)}{3}\text{.} \end{array}$$

The force balance equation based on the HD model can be represented by the case J=1 in Eq. (9),

(11) $$\begin{array}{c} f_{\text{cap},\text{HD}}+f_{\text{visc},\text{HD}}=0\text{.} \end{array}$$

We now compare Eq. (11) of the HD model with Eq. (9) of the RC model. We include the forces from the HD model for the comparison. In the HD model, when J>1, the effect of capillary force on the viscous force is smaller than that in the RC model, and, conversely, when J<1, the effect is greater than that in the RC model.

The conversion equation J(ε) is defined such that ζ(ε) is included in the numerator. Thus, J(ε) depends on ε, but not on time t or distance l. The approximation of ζ(ε) is employed to analyze J(ε). Various approximations of ζ(ε) were proposed [[23], [24], [25]]. Here, the infinite series in ζ(ε) was approximated by summing up to a finite N, as the range of valid ε in the approximation depends on N

(12) $$\begin{array}{c} \zeta \left(\varepsilon \right)∽1-6\varepsilon \sum_{k=0}^{N}{\left(\frac{2}{\left(2k+1\right)\pi }\right)}^{5}\;\tanh\left[\frac{\left(2k+1\right)\pi }{2\varepsilon }\right]\text{.} \end{array}$$

We now substitute the approximation Eq. (12) into Eqs. (7), (10)

(13) $$\begin{array}{c} K_{2,N}\left(\varepsilon \right)=\frac{2}{3}\pi \varepsilon \times \left\{1-6\varepsilon \sum_{k=0}^{N}{\left(\frac{2}{\left(2k+1\right)\pi }\right)}^{5}\;\tanh\left[\frac{\left(2k+1\right)\pi }{2\varepsilon }\right]\right\}\text{.} \end{array}$$

(14) $$\begin{array}{c} J_{N}\left(\varepsilon \right)=\frac{2{\left(1+\varepsilon \right)}^{2}}{3}\times \left\{1-6\varepsilon \sum_{k=0}^{N}{\left(\frac{2}{\left(2k+1\right)\pi }\right)}^{5}\;\tanh\left[\frac{\left(2k+1\right)\pi }{2\varepsilon }\right]\right\}\text{.} \end{array}$$

**Fig. 1:**
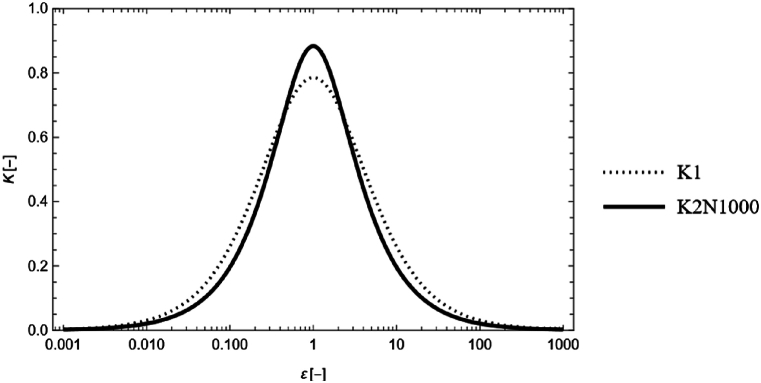
Single logarithmic graph of K when 0.001<ε<1000. $K_{1}$ and $K_{2}$ are related to the capillary and viscous forces, respectively.

As illustrated in Fig. 1, both $K_{1}$ and $K_{2}$ yield values that are less than 1. $K_{2}$ is given for N=1000. This indicates that compared with the RC model, the HD model underestimates the magnitude of each force.

**Fig. 2:**
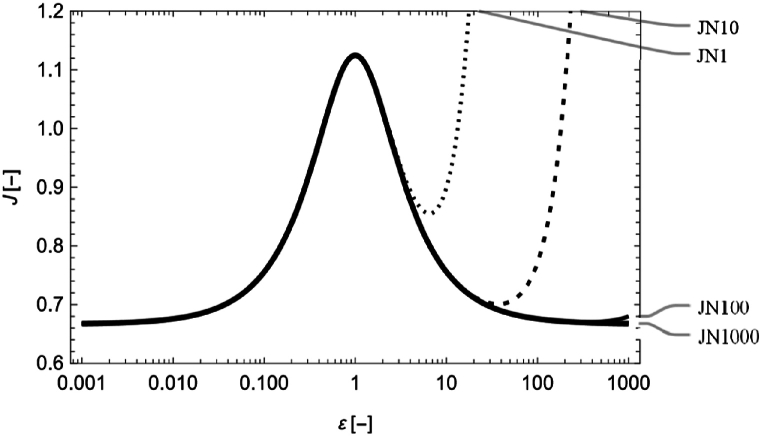
Single logarithmic graph of J when 0.001<ε<1000, where JN1, JN10, JN100, and JN1000 indicate $J_{N}\left(\varepsilon \right)$ at N=1,10,100, and 1000, respectively.

For J(ε) representing the relationship between HD and RC models expressed by Eq. (10), the resulting J−ε graph is illustrated in Fig. 2. The graph is plotted for each of the four values of N (1,10,100, and 1000). To evaluate the approximation for different values of N, the duality for the interchange of ε and 1/ε (ε-duality) was employed. When the surface conditions and contact angle are the same throughout a rectangular channel, the depth *h* and width *w* of the cross-section can be interchanged without affecting the flow. The interchange of h and w corresponds to that of ε and 1/ε. The rectangular channel model is invariant with respect to the interchanging of *ε* and 1/ε, and it is considered to have ε-duality. The validity of the approximation of ζ can be discussed in terms of whether ε-duality holds for rectangular channels. A single logarithmic graph, as shown in Fig. 2, is used to elucidate the ε-duality. This duality is determined by the symmetry about the ε=1 axis in a single logarithmic graph. When ε<1, $J_{N}$ is the same for N=1,10,100, and 1000. Conversely, when ε>1, each value of $J_{N}$ exhibits a different shape. For N=1, the range of ε-duality adaptation is narrow because of the rough approximation, whereas the range is wide for N=1000. Considering ε-duality, the larger the value of N, the wider the range of useable ε in the approximation. The approximation of $J_{N\;}\left(N=1000\right)$ is satisfied in the range 0.001<ε<1000, and the following discussion is based on this approximation.

#### 2.2.2 Flow distance solution

Considering the flow distance solution on the horizontal plane, from Eq. (3), the flow distance solution in the HD model is

(15) $$\begin{array}{c} l_{\text{HD},h}={\left(\frac{h\gamma \;\cos\;\theta }{2\mu \left(1+\varepsilon \right)}t\right)}^{\frac{1}{2}}\text{.} \end{array}$$

From Eq. (4), the flow distance solution in the RC model is

(16) $$\begin{array}{c} l_{\text{RC},h}={\left(\frac{\left(1+\varepsilon \right)\zeta \left(\varepsilon \right)h}{3}\frac{\gamma \;\cos\;\theta }{\mu }t\right)}^{\frac{1}{2}}\text{.} \end{array}$$

In comparing the two flow distance solution of the two models,

(17) $$\begin{array}{c} l_{\text{RC},h}\left(t\right)={\left(\frac{h\gamma \;\cos\;\theta }{2\mu \left(1+\varepsilon \right)}Jt\right)}^{\frac{1}{2}}=l_{\text{HD},h}\left(Jt\right) \end{array}$$

is satisfied. Converting the *t* part of the flow distance in the HD model $l_{\text{HD}}$ to Jt yields a result that is equal to the flow distance in the RC model $l_{\text{RC}}$.

**Fig. 3:**
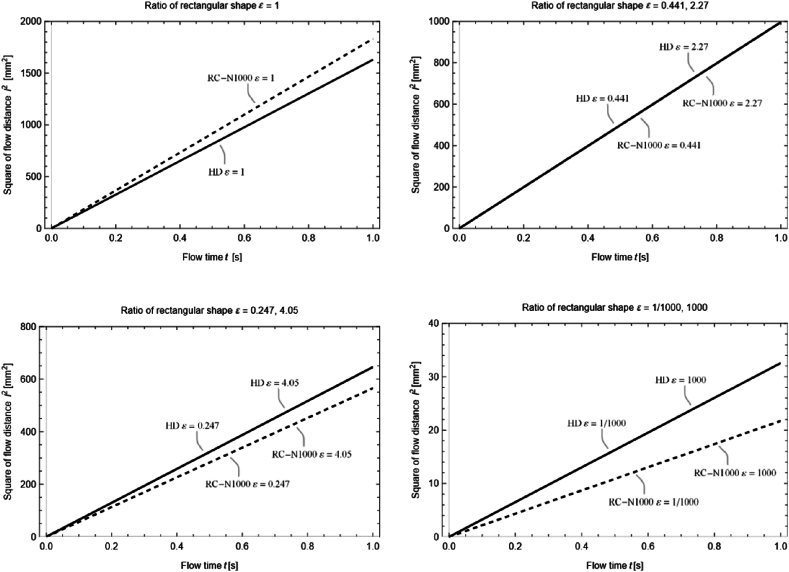
Relationship between the square of flow distance $l^{2}$ and flow time t at ε=1 (top left), ε=0.441 and 2.27 (top right), ε=0.247 and 4.05 (bottom left), and ε=1/1000 and 1000 (bottom right). The solid and dashed lines represent the HD and RC-N1000 (RC-N at N=1000) models, respectively. The graph used the following parameters; viscosity of water $\mu =1.00\times 10^{-3}\;\text{Pa}\cdot s$, density of water $\rho =9.98\times 10^{2\;}\text{kg}/m^{3}$, surface tension of water $\gamma =7.27\times 10^{-2\;}{\text{kg}/s}^{2}$, and contact angle θ=26.3°.

Several noteworthy values of $\varepsilon ,{\;J}_{N\;}\left(N=1000\right)$ and the relationships between $l_{\text{HD}}$ and $l_{\text{RC}}$ and $K_{1}$ and $K_{2}$ are summarized in Table 1. The resulting $l^{2}-t$ graphs produced using both the HD and RC models are presented in Fig. 3. The RC model based on the approximation of Eq. (12), referred to as the RC-N model, contains an upper bound N on the summation of the infinite series, and RC-N1000 indicates the RC-N model at N=1000. For these results, 0.001<ε<1000, and 0.668<J<1.125. The value of J indicates the influence of the capillary and viscous forces as they are converted to the HD model. At J=1, i.e., ε=0.441 and 2.27, the capillary and viscous forces are converted at the same rate; hence, the HD and RC models coincide. This is evident in the $l^{2}-t$ graph of the two models (Fig. 3, top right). At ε=1, where h and w are equal, J=1.125, which is +12.5% with respect to J=1. Similarly, at J=0.875, which is −12.5% with respect to J=1, ε=0.247 and 4.04. This shows that, for 0.247<ε<4.04, J is in the range of ±12.5%. When J>1, the effect of the viscous force is larger than that of the capillary force in the conversion to the HD model. Therefore, when 0.441<ε<2.27, i.e., the cross-section of the flow channel resembles a square, $l_{\text{HD}}$ is smaller than $l_{\text{RC}}$ (Fig. 3, top left). However, when J<1, the effect of the capillary force is larger than that of the viscous force in the conversion to the HD model. Therefore, when ε<0.441 and 2.27<ε, i.e., as the cross-section of the flow channel becomes elongated, $l_{\text{HD}}$ becomes larger than $l_{\text{RC}}$ (Fig. 3, bottom left and right).

**Table 1:** Relationship between $\varepsilon ,J_{N\;}\left(N=1000\right),l\text{,}$ and K, where *l* represents the relationship between the values of $l_{\text{HD}}$ and $l_{\text{RC}}$ and K represents the relationship between the values of $K_{1}$ and $K_{2}$.

ε	0.001	0.247	0.441	1.000	2.27	4.05	1000
J	0.668	0.875	1.000	1.125	1.000	0.875	0.668
l	$l_{\text{HD}}>l_{\text{RC}}$		$l_{\text{HD}}=l_{\text{RC}}$	$l_{\text{HD}}<l_{\text{RC}}$	$l_{\text{HD}}=l_{\text{RC}}$	$l_{\text{HD}}>l_{\text{RC}}$	
K	$K_{1}>K_{2}$		$K_{1}=K_{2}$	$K_{1}<K_{2}$	$K_{1}=K_{2}$	$K_{1}>K_{2}$

## 3 Experiments

**Fig. 4:**
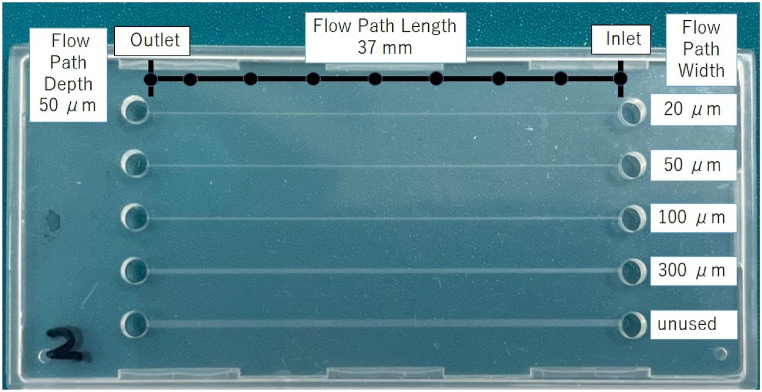
Flow channels. The channel widths are 20,50,100, and 300μm from the top channel. The bottom channel is inaccessible owing to an obstruction in the midsection.

Experiments were conducted using aqueous ethanol solutions in rectangular microfluidic channels (Richell Corporation, Natural Flow Chip Type III). Aqueous ethanol solutions were made from ethanol (HAYASHI PURE CHEMICAL IND., LTD., Ethanol (99.5), purity: 99.5 %) and pure water (YONEYAMA YAKUHIN KOGYO CO., LTD., Pure Water, purity: 100 %). The microfluidic channels were made of a special resin based on polypropylene and TPE. Fig. 4 is the flow channel image used for the experiment. Four different channels with depth h=50μm, channel length L=37mm, and widths w=20,50,100 and 300μm, respectively, were used, as listed in Table 2. The values of ε and J for each channel width are also presented in Table 2. The channel widths are 20,50,100, and 300μm from the top channel in Fig. 4. However, the bottom channel was not used owing to an obstruction in the midsection. Aqueous ethanol solutions with different concentrations were used as the working fluid. The concentrations of the solutions were 100wt%,80wt%,60wt% and 40wt%. Under the experimental conditions, the temperature was around 20°C and the pressure was a constant atmospheric pressure at the inlet/outlet. The effects of temperature, pressure, and humidity are briefly discussed below. Physical properties, such as viscosity and surface tension, depend on temperature. The relationship between flow distance and flow time changes with pressure since pressure acts as a force with respect to flow. In these experiments, the same atmospheric pressure is applied at the inlet/outlet and cancels each other out, so the pressure is assumed to have no effect. Humidity is assumed to have no effect on the flow equation. The flow was recorded from the top of the meniscus, and the flow time at the meniscus was measured at 0 (inlet), 5, 10, 15, 20, 25, 30, 35, and 37 (outlet) mm (black circle position) from the channel inlet. The experiments were repeated eight times under each condition.

**Table 2:** Structure of rectangular flow channels.

w[μm]	20	50	100	300
ε[−]	2.5	1.0	0.5	0.167
J[−]	0.978	1.12	1.03	0.812

## 4 Results and discussion

### 4.1 Flow models considerations based on experimental results

In our experiments, the slope of the graph was determined by squaring of the liquid meniscus travel distance $l^{2\;}\left[{\text{mm}}^{2}\right]$ on the horizontal axis and the flow time t[s] on the vertical axis. As the values on the horizontal and vertical axes have a proportional relationship, this slope was used as the measured value of $\frac{t}{l^{2}}$. Eq. (4) was then transformed as follows, based on which a graph was drawn.

(18) $$\begin{array}{c} \frac{1}{r_{\text{HD}}J}=\frac{\gamma \;\cos\;\theta }{2\mu }\frac{t}{l^{2}} \end{array}$$

**Fig. 5:**
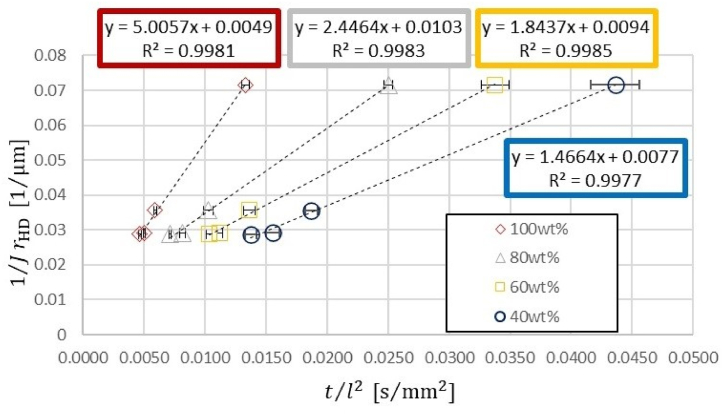
Graph with the experimental value $t/l^{2}$ on the horizontal axis and flow path structure value based on the RC model $1/J\;r_{\text{HD}}$ on the vertical axis.

**Fig. 6:**
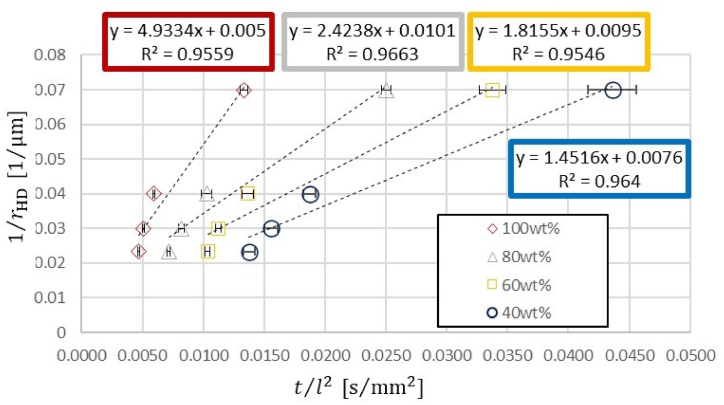
Graph with the experimental value $t/l^{2}$ on the horizontal axis and flow path structure value based on the HD model $1/r_{\text{HD}}$ on the vertical axis.

Here, the left-hand side contains the HD value $r_{\text{HD}}$ and values related to the channel structure such as the conversion formula *J*, while the right-hand side contains the physical properties of the flowing solution $\frac{\gamma \;\cos\;\theta }{2\mu }$ and the experimental value $\frac{t}{l^{2}}$. Fig. 5 and Table 3 illustrate the experimental value $\frac{t}{l^{2}}$ on the horizontal axis and the flow path structure value $\frac{1}{r_{\text{HD}}J}$ on the vertical axis. The relationship given by Eq. (18) is confirmed because the plot of each ethanol solution lies on a linear approximate straight line. The slope of this linear approximate straight line is the value $\frac{\gamma \;\cos\;\theta }{2\mu }$ for the physical properties of the flowing solution. The value of the slope decreases as the concentration of the ethanol solution decreases. If only the inverse of the HD was taken on the vertical axis without using the conversion equation J, it will be represented as illustrated in Fig. 6 and Table 3. Coincidentally, the linear approximate straight line for each aqueous ethanol solution plot is almost identical to that in Fig. 5. Plots that lie on the linear approximate straight line can be considered as J=1, and the two plots with channel widths w=20 and 100μm resemble the shape of this linear approximate straight line. At w=50μm channel width, where J>1, the plot is above the line, and at *w* = 300 μm channel width, where *J <* 1, it is below the line. The deviation of the plot from the linear approximate straight line represents the discrepancy that arises from Eq. (18) of the HD model.

**Table 3:** Representing the ratio of the flow time to the square of the flow distance of each aqueous ethanol solution with respect to $1/J\;r_{\text{HD}}$ and $1/r_{\text{HD}}$ at each channel cross-section.

$1/Jr_{\text{HD}}$ $\left[\mu m^{-1}\right]$	$1/r_{\text{HD}}$ $\left[\mu m^{-1}\right]$	100 wt%$t/l^{2}\;\left[s/{\text{mm}}^{2}\right]$	80 wt% $t/l^{2}\;\left[s/{\text{mm}}^{2}\right]$	60 wt% $t/l^{2}\;\left[s/{\text{mm}}^{2}\right]$	40 wt% $t/l^{2}\;\left[s/{\text{mm}}^{2}\right]$
0.0287	0.0233	0.0047±0.0001	0.0072±0.0001	0.0104±0.0002	0.0138±0.0005
0.0291	0.0300	0.0051±0.0001	0.0082±0.0002	0.0112±0.0002	0.0156±0.0005
0.0356	0.0400	0.0059±0.0001	0.0103±0.0004	0.0137±0.0004	0.0187±0.0005
0.0716	0.0700	0.0133±0.0003	0.0251±0.0004	0.0338±0.0010	0.0436±0.0020

We investigated rectangular channel solutions with hydraulic diameters for 0.001<ε<1000 and found that the misalignment ranges from −33.2% to 12.5% for *J*. For a square cross-section (ε=1), J=1.125 and the deviation is 12.5%, which is considered the maximum range of adaptable deviation. It is shown that *J* is in the range of ±12.5% for 0.247<ε<4.04. Thus, in this study, a useful range of ε for the hydraulic diameter model is shown with respect to a square cross-section.

### 4.2 Comparison of other approximate models

**Fig. 7:**
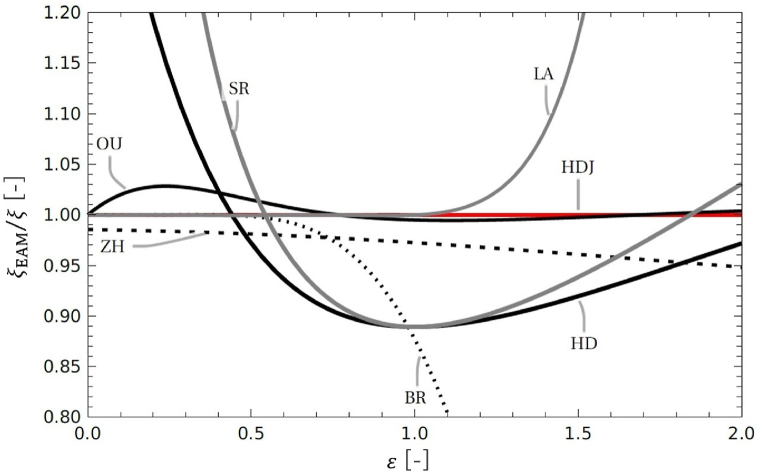
Comparison of each approximate model to RC model with $\zeta_{\text{EAM}}/\zeta$ plotted on the vertical axis and the aspect ratio ε on the horizontal axis.

Based on Eq. (4), we state the expression corresponding to ζ(ε) in the flow equations of each approximate model of rectangular channels and compare it with ζ(ε) of the RC model. In Fig. 7, $\zeta_{\text{EAM}}$ (EAM indicates each approximate model) is set to correspond to ζ in Eq. (4) for each approximate model; the ratio $\zeta_{\text{EAM}}/\zeta$ is plotted on the vertical axis and aspect ratio *ε* on the horizontal axis. Some approximations of exact solutions for rectangular-type channels have been shown to be useful. Bruus [24] and Zhu et al. [25] presented approximations assuming that ε is less than 1. Ouali et al. [23] introduced an approximation that is used with *ε* values ranging between 0 and 2. For comparison with these approximate models, the graphs in Fig. 7 are plotted in the range 0<ε<2.

In the HD model, the ratio $\zeta_{\text{EAM}}/\zeta$ is

(19) $$\begin{array}{c} \zeta_{\text{HD}}=\frac{3}{2{\left(1+\varepsilon \right)}^{2}}\text{.} \end{array}$$

The relationship between $\zeta_{\text{HD}}/\zeta$ and J is inverse. If we return from the HD model to the exact solution of the RC model using the conversion function J, we obtain

(20) $$\begin{array}{c} \zeta_{\text{HDJ}}=\frac{3}{2{\left(1+\varepsilon \right)}^{2}}J\text{.} \end{array}$$

In this model, $\zeta_{H\text{DJ}}/\zeta =1$ is satisfied.

We introduce three models as approximations of Eq. (5) used in the RC model of exact solutions. According to Bruus [24], for ε<1, the approximation is

(21) $$\begin{array}{c} \zeta_{\text{BR}}=1-0.630\varepsilon \text{.} \end{array}$$

According to Zhu et al. [25], for ε<1, we have

(22) $$\begin{array}{c} \zeta_{\text{ZH}}=\frac{96}{\pi^{4}}\left\{1-\frac{2\varepsilon }{\pi }\tanh\left(\frac{\pi }{2\varepsilon }\right)\right\}\text{.} \end{array}$$

For ε→0, it asymptotically approaches $\zeta_{\text{ZH}}/\zeta \to 0.986$. According to Ouali et al. [23], for ε<2,

(23) $$\begin{array}{c} \zeta_{\text{OU}}=\frac{1}{1-0.362374\varepsilon +1.020980\varepsilon^{2}}\text{.} \end{array}$$

For 0<ε<2, $\zeta_{\text{OU}}/\zeta$ takes a value close to 1. As ε→1000 and ε increases, $\zeta_{\text{OU}}/\zeta \to 0.980$. This approximation shows a good approximation over a wide range of $0.980<\zeta_{\text{OU}}/\zeta <1.028$.

As other approximate models for rectangular channels, Bahrami et al. [32] and Muzychka and Yovanovich [33] proposed an approximation method that uses the square root of the cross-sectional area as the length for rectangular channels.

(24) $$\begin{array}{c} \zeta_{\text{SR}}=\frac{3}{4\left(1+\varepsilon \right)\varepsilon^{\frac{1}{2}}} \end{array}$$

For 0<ε<2, the behavior is similar to that of the HD model. Berthier et al. [34] proposed a model that derives information on the cross-section of a channel using the friction length. Using this lambda's method, we obtain

(25) $$\begin{array}{c} \zeta_{\text{LA}}=\frac{1}{{\left(1+\varepsilon \right)}^{2}\left(1-1.3553\varepsilon +1.9467\varepsilon^{2}-1.7012\varepsilon^{3}+0.9564\varepsilon^{4}-0.2537\varepsilon^{5}\right)}\text{.} \end{array}$$

It takes a value close to 1 for 0<ε<1 and a positive value for 0<ε<2.

The HD model resembles existing models of Bahrami et al. [32] and Muzychka and Yovanovich [33]. The approximate models of Ouali et al. [23] and Zhu et al. [25] show a better approximation for a wider range of aspect ratio *ε* than the HD model. The approximate models of Bruus [24] and Berthier et al. [34] are limited in the range of aspect ratio ε. Conversely, the approximate models of Ouali et al. [23] and Zhu et al. [25] are valid for a wider range of aspect ratio ε than the HD model. The deviation between the HD and the RC models is larger than other approximate models in the range of 0<ε<2.

## 5 Conclusions

We proposed a conversion equation *J*(*ε*), which depends on *ε*, to convert the solutions of the HD to RC (based on the exact solution of the balance of forces in steady flow) models for rectangular microchannels. We employed the *ζ*(*ε*) approximation to analyze *J*(*ε*). The advantage of the J(ε) conversion equation is that if the value of the aspect ratio of rectangular cross-section ε is known, the value of J(ε) is known, so when the flow in a rectangular channel is calculated using the HD model, it is possible to know how much the deviation from the RC model (exact solution) is by J(ε). Simultaneously, the HD model calculation can be easily converted to the RC model flow calculation.

To compare the HD model with other approximate models, the equation corresponding to ζ(ε) in the flow equations of each approximate model for rectangular channels is written based on Eq. (4), and the ratio of ζ(ε) of RC model. Fig. 7 is the ratio for 0<ε<2. The results show that the HD model is dependent on the aspect ratio ε, and the deviation is larger than other approximate models. Thus, it is possible to obtain a more accurate flow based on the HD model by modifying it with the conversion equation J.

When the flow channel cross-section was close to a square, $l_{\text{HD}}$ was smaller than $l_{\text{RC}}$. In contrast, when the cross-section was elongated, $l_{\text{HD}}$ was greater than $l_{\text{RC}}$. The two models coincided when ε=0.441 and 2.27. When 0.247<ε<4.04, J(ε) was within a deviation of ±12.5%. Rectangular flow experiments using ethanol solution confirmed the difference between the RC and HD models, represented by J. When J=1, the two models converge. The HD model lost its accuracy as the value of J increased or decreased from 1. The plot illustrated in Fig. 6 deviates from the linear approximate straight line; this corresponds to an experiment in a channel with a certain aspect ratio where the value of J deviates from 1.

In future studies, we shall investigate cases where the contact angle is different for each side of the rectangular channel. Moreover, we shall compare it with the model based on the square root of the channel's cross-section area as the characteristic length scale for a rectangular channel.

## CRediT authorship contribution statement

**Koichiro Kobayashi:** Writing – review & editing, Writing – original draft, Project administration, Methodology, Investigation, Formal analysis, Data curation, Conceptualization. **Kenji Sakamoto:** Writing – review & editing, Project administration, Methodology, Investigation, Data curation, Conceptualization.

## Declaration of competing interest

The authors declare that they have no known competing financial interests or personal relationships that could have appeared to influence the work reported in this paper.

## Data Availability

Data will be made available on reasonable request.
